# TREM-1-dependent M1 macrophage polarization restores intestinal epithelium damaged by DSS-induced colitis by activating IL-22-producing innate lymphoid cells

**DOI:** 10.1186/s12929-019-0539-4

**Published:** 2019-06-12

**Authors:** Fu-Chen Yang, Po-Yuan Chiu, Yun Chen, Tak W. Mak, Nien-Jung Chen

**Affiliations:** 1 Institute of Microbiology and Immunology, School of Life Sciences, National Yang-Ming University, No.155, Sec.2, Linong Street, Taipei, Taiwan; 2 Department of Surgery, Far Eastern Memorial Hospital, New Taipei City, Taiwan; 3 The Campbell Family Institute for Breast Cancer Research, Ontario Cancer Institute, University Health Network and Department of Medical Biophysics, University of Toronto, Toronto, Ontario M5G 2C1 Canada; 4 Cancer Progression Research Center, National Yang-Ming University, Taipei, Taiwan

**Keywords:** Triggering expressed on myeloid cells-1, Inflammatory macrophages, IL-22, Group 3 innate lymphoid cells, Colitis

## Abstract

**Background:**

Triggering receptor expressed on myeloid cells-1 (TREM-1) is highly expressed on macrophages in inflamed intestines and reportedly promotes inflammatory bowel disease (IBD) by augmenting pro-inflammatory responses. To study the mechanism mediated by TREM-1 on macrophages, we generated an independent TREM-1 deficient mouse.

**Methods:**

Acute colitis was induced in C57BL/6 and TREM-1-deficient mice by the administration of dextran sodium sulfate (DSS). Colonic lamina propria immune cell composition and cytokines were analyzed. An innate lymphoid cell (ILC) co-culture experiment with macrophages was used to analyze IL-22 levels. Exogenous IL-22 and TREM-1-expressing macrophages were supplied to TREM-1-deficient mice for examining their effects on intestinal barrier integrity.

**Results:**

In inflamed colons, TREM-1 loss compromised the activation of ILC3 and their production of IL-22, which is required for intestinal barrier integrity. ILC3-mediated IL-22 production depends on IL-1β secreted by M1-polarized macrophages, and we found that TREM-1 deficiency results in a decreased number of IL-1β producing-M1 macrophages in colons exposed to DSS. Accordingly, DSS-mediated damage was ameliorated by supplying exogenous IL-22 and TREM-1-expressing macrophages to TREM-1-deficient mice.

**Conclusions:**

TREM-1 plays a crucial role in regulating IL-22 production by ILC3 through modulating M1-macrophage polarization during DSS-induced acute colitis.

## Background

Inflammatory bowel disease (IBD) is characterized by chronic inflammation of the intestine and comprises two major subtypes: ulcerative colitis (UC) and Crohn’s disease (CD) [1, 2]. The inflammation associated with CD can affect any section and any tissue layer of the gastrointestinal (GI) tract. In contrast, the inflammation associated with UC is restricted to the mucosal surface of the intestine and can extend in a continuous manner from the start of the colon to the proximal rectum. Both UC and CD are driven by alterations to the composition of local immune cell compartments and exaggerated immune responses to microbial products [3].

Macrophages play a major role in the body’s first line of defense against foreign antigens and control the barrier functions of the epithelial layers in the small intestine and colon [4]. They also modulate intestinal steady-state homeostasis and regulate intestinal inflammation. Macrophages thus most often play a protective role against the development of acute colitis [5]. Based on their state of activation, most macrophages can be grouped into two subtypes: classically activated and pro-inflammatory (M1) macrophages and alternatively activated and anti-inflammatory (M2) macrophages. In the early stages of intestinal barrier disruption, macrophages are stimulated and polarized towards the M1 phenotype, leading to the elimination of pathogens by the production of a massive amount of cytokines and nitric oxide (NO); in the late stages of the inflammatory response, macrophages may become polarized towards the M2 phenotype to trigger wound healing [6]. However, the distinct functions of M1 macrophages and M2 macrophages in this context are not clear, and the various receptors, ligands and cytokines in the local microenvironment that govern intestinal barrier integrity have yet to be completely investigated.

The TREM family of molecules is a group of pattern recognition receptors (PRRs) belonging to the surface immunoglobulin receptor superfamily. TREM-1 was the first TREM family member to be characterized, was found to be constitutively expressed by activated macrophages and resting neutrophils and is upregulated on these cells upon their exposure to microbial products, prostaglandin E2 (PGE2), or granulocyte macrophage colony-stimulating factor (GMCSF) [7–9]. A pathogenic role for TREM-1 in colitis was suggested by the finding that both soluble TREM-1 (sTREM-1) in serum and TREM-1 mRNA in inflamed colonic tissue were elevated in patients with IBD [10]. Accordingly, the effects of TREM-1 inhibition, achieved by the administration of an antagonistic peptide to mice, were examined following the induction of DSS-induced colitis or colitis-associated carcinogenesis (CAC). It was found that interference with TREM-1 activity diminished pro-inflammatory cytokine production in the colon and reduced the proliferation of intestinal epithelial cells [11, 12].

An intact epithelial barrier is a critical component of intestinal immunity. DSS-induced colitis is a chemical process that leads to superficial ulceration of the intestinal epithelium with subsequent increases in intestinal luminal bacterial translocation and infiltration of acute inflammatory immune cells [13, 14]. Regeneration of the intestinal epithelium is critical for barrier restoration after tissue injury, and various reports have indicated that IL-22 plays a key role in this regeneration, thereby protecting the colon against acute colitis [15–18]. IL-22 is produced mainly by T helper type 17 (Th17) cells and group 3 innate lymphoid cells (ILC3), which are a subset of ILCs in the gut which express a novel RAR-related orphan receptor gamma t (RORγt) [19]. ILC3 cells reside in the intestinal lamina propria and direct innate immune responses to tissue damage through the rapid activation of epithelial signal transducer and activator of transcription-3 (STAT3). In response to luminal bacteria translocation to a mucosal surface, intestinal macrophages regulate the tissue injury-induced IL-1β responses, which maintain and enhance ILC3 cells’ ability to produce large amounts of powerful mediators, including IL-22, that are critical for maintaining the integrity of the intestinal epithelial barrier [17, 20, 21] and preserving intestinal stem cells [20, 22]. In a DSS-induced mouse model of colitis, IL-22 deficiency exacerbates colitis pathogenesis and alters the colonic microbiota to be more colitogenic compared to the control group [15, 18, 23]. Interestingly, recent evidence has revealed a potent role for lamina propria macrophages in integrating microbial signals to regulate colonic ILC3 activation in IBD [24, 25].

To investigate whether the phenotype of TREM-1-mediated macrophages regulates IBD pathogenesis, we examined the effects of DSS-induced colitis on an in-house generated independent line of TREM-1-deficient (TREM-1 KO) mice [7]. Unexpectedly, we discovered a protective role for TREM-1 in regulating intestinal epithelial integrity. TREM-1 increases IL-1β production by M1 macrophages during DSS-induced colonic inflammation and thereby influences ILC3-mediated IL-22 production. Moreover, our data demonstrate that intestinal tissue damage from DSS-induced colitis can be alleviated by supplying exogenous IL-22 or WT macrophages to TREM-1 KO mice.

Taken together, our results revealed the dual nature of TREM-1’s involvement in IBD through its modulation of M1 macrophage differentiation and function: promoting inflammatory cytokine production on the one hand but activating IL-22-producing ILC3 cells on the other.

## Methods

### Mice

The chimeric TREM-1 knockout mice were generated in the 129/SvJ and C57BL/6 hybrid background as described [26] and have been backcrossed with C57BL/6 J mice for more than 10 times to produce heterozygous *Trem-1*^+/−^ mice. The heterozygotes were intercrossed to generate homozygous *Trem-1*^−/−^ mice. WT mice and conventional TREM-1 knockout (B6.129P2-*Trem-1*^tm1Mak^) [7] mice of the C57BL/6 J genetic background were bred and maintained under specific pathogen-free conditions (but positive for *Helicobacter spp.*) in the animal center of National Yang-Ming University in accordance with Institutional Animal Care and Use Committee guidelines. Age-matched males (8–10 weeks old) were used for experiments.

### DSS-induced colitis

To avoid potential differences in gut microflora between WT and TREM-1 deficient mice, Male WT and TREM-1 deficient mice were co-housing before weaning, and their cages from each group were exchanged at least a week prior to the starting of experiments to minimize the variations generated by environment. Acute colitis was induced in WT and TREM-1 KO mice by administration of 3% DSS (36,000–50,000 MW; MP Biomedical) in drinking water for 5 days, followed by 4 days of regular tap water. Mice were euthanized at day 7 or day 9 after the initial administration of DSS. For rescue experiments, mice were intraperitoneally injected every other day (starting on 1 day before DSS treatment (day − 1) and ending on day 5 of the protocol) with either 500 ng recombinant murine IL-22 (Peprotech, Rocky Hill) in 100 μl PBS, or with PBS alone. For macrophage transfer experiments, mice were intraperitoneally injected with either 2*10^6^ cells in 200 μl PBS or with PBS alone on day 5.

### Pathological and histological analyses

Pathological changes were evaluated using a method that was modified from the standard UCDAI scoring system [27] and based on following parameters: a decrease in body weight relative to initial weight (0–3%, score of 0; 4–10%, 1; 11–20%, 2; 21–30%, 3; and 31–40%, 4); stool viscosity (normal, score of 0; soft, 1; loss, 2; and diarrhea, 3); and rectal bleeding (normal, score of 0; streak of blood with stools, 1; and obvious blood with stools, 2). Colon length was measured from the anus to the adjoining point of the cecum and the small intestine. To assess histological alterations in the distal colon and rectum, samples of these tissues were fixed in 4% paraformaldehyde, embedded in paraffin, and stained with hematoxylin and eosin (HE; Muto Pure Chemicals). Images were acquired using a Nikon Eclipse 80i microscope. Histopathology was graded using a previously established scoring system based on the following parameters: inflammation severity (none, score of 0; moderate, 1; substantial, 2; and severe, 3); depth of injury (none, score of 0; mucosal, 1; mucosal and submucosal, 2; and transmural, 3); crypt damage (none, score of 0; basal one-third damaged, 1; basal two-thirds damaged, 2; only surface epithelium intact, 3; and entire crypt epithelium lost, 4); and percentage of area involved (none, score of 0; 1–25%, 1; 26–50%, 2; 51–75%, 3; and 76–100%, 4) [28]. To visualize goblet cells in epithelial layer, paraffin-embedded tissue samples were stained with alcian blue and periodic acid-Schiff (AB-PAS; Sigma-Aldrich). Images were acquired using a Nikon Eclipse 80i microscope.

### Gut permeability assay

Mice received intrarectal injection of 50 μl fluorescein isothiocyanate (FITC)–conjugated dextran (25 mg/ml; mean molecular weight 4000; Sigma-Aldrich) using a round-tip feeding needle. Mice were sacrificed 30 min later, and the FITC-dextran concentration in the plasma was determined using a fluorescent microplate reader (BioTek Synergy HT) and a standard curve generated by serial dilution [29].

### RNA extraction and real-time PCR analysis

Distal colonic tissue samples (0.5 cm in length) were suspended in TRIzol reagent (Invitrogen) and homogenized with MagNA Lyser Green Beads (Roche Life Science). RNA was extracted using an RNA Purification Kit (Qiagen) following the manufacturer’s instructions. Complementary DNA (cDNA) was generated using a High Capacity cDNA Reverse Transcription Kit (Applied Biosystems). Quantitative real-time PCR analysis was performed on an Mx3000P™ instrument (Strategene) using KAPA SYBR FAST qPCR Master Mix (Kapa Biosystems). Sequences of the PCR primer sets used are listed in Table 1.

**Table 1:** Sequences of primers used in this study

Gene	5′ to 3′ end	Sequence
GAPDH	F	GCATCCACTGGTGCTGCC
	R	TCATCATACTTGGCAGGTTTC
TREM-1	F	GTCTCAGAAGTCAAAGCTGC
	R	GTCTGGTAGTCTCTGCCAAG
TNFα	F	CCTCACACTCAGATCATCTTC
	R	CGGCTGGCACCACTAGTTG
IL-6	F	GCCTTCCCTACTTCACAAGT
	R	GAATTGCCATTGCACAACTCT
IFNγ	F	CTTCCTCATGGCTGTTTCTG
	R	TGTCACCATCCTTTTGCCAG
IL-1β	F	TTGAAGAAGAGCCCATCCTC
	R	CAGCTCATATGGGTCCGAC
IL-17a	F	GCTTCATCTGTGTCTCTGATG
	R	GCGCCAAGGGAGTTAAAGAC
IL-22	F	TCCGAGGAGTCAGTGCTAA
	R	AGAACGTCTTCCAGGGTGAA
IL-23a	F	GCCTGGAACGCACATGCAC
	R	CCTTTGCAAGCAGAACTGGC
GMCSF	F	GAACCTCCTGGATGACATGC
	R	CAGTCCGTTTCCGGAGTTG
iNOS	F	CATTCTACTACTACCAGATCG
	R	GCAAAGAACACCACTTTACC
IL-12a	F	ACATGGTGAAGACGGCCAG
	R	GAAGTCTCTCTAGTAGCCAG
Arg-1	F	GGGTGGAGACCACAGTCTG
	R	AGTGTTGATGTCAGTGTGAGC
YM-1	F	TTATCCTGAGTGACCCTTCTAAG
	R	TCATTACCCTGATAGGCATAGG
IL-10	F	ATGCAGGACTTTAAGGGTTAC
	R	CCTGAGGGTCTTCAGCTTC

### Differentiation of bone marrow-derived macrophages

Total bone marrow cells were isolated from mice and induced to differentiate as previously described [7]. Briefly, bone marrow cells were incubated for 5 days with 20 ng/ml GMCSF and then plated in Gibco RPMI medium 1640 (Thermo Fisher Scientific) containing 10% fetal bovine serum (FBS; Biological Industries) and 1% antibiotics (100 mg/ml penicillin/streptomycin; Biological Industries). Adherent cells were deemed to be GMCSF-differentiated bone marrow-derived macrophages (GM-BMDMs). For activation experiments, GM-BMDMs were stimulated with 5 ng/ml lipopolysaccharide (LPS; Invivogen), or 5 ng/ml LPS plus plate-bound 8 μg/ml anti-TREM-1 agonist antibody (MA5–16765; Thermo) or isotype antibody (Rat IgG2aκ; eBioscience), or 45 ng/ml IFNγ plus 20 ng/ml LPS, or 20 ng/ml IL-4 plus 20 ng/ml IL-13 (Peprotech).

### Isolation of colonic lamina propria (cLP) cells

Mouse colon was cut into 0.5 cm pieces and incubated for 30 min at 37 °C in calcium- and magnesium-free Dulbecco and Phosphate-Buffered Saline (DPBS; Thermo Fisher Scientific) supplemented with 1 mM EDTA. Gentle stirring was applied to remove intestinal epithelial cells and the supernatant was discarded. The remaining colonic fragments were incubated in RPMI medium containing 1 mg/ml Collagenase D (Roche Life Science), 100 μg/ml DNase I (Roche Life Science), and 2% FBS. Gentle stirring was applied for 60 min at 37 °C. After centrifuge the supernatant was discarded, the wash repeated, and the remaining cells were filtered through a 40 μm Nylon strainer (BD Biosciences) to obtain a single cell suspension of cLPs. To isolate colonic macrophages, cLPs were stained with anti-mouse F4/80 Microbeads (Miltenyl Biotec). F4/80^+^ macrophages were isolated from cLPs using an AutoMACS Pro Separator system (Miltenyl Biotec).

### FACS analysis

Isolated cLP cells were suspended in PBS containing 2% FBS and subjected to flow cytometric analysis as described previously [30]. Anti-CD16/32 antibody (from clone 2.4G2-conditioned medium) was used to block non-specific binding to Fcγ receptors before surface staining. Dead cells were excluded from the analysis by staining with the Zombie Red Fixable viability kit (BioLegend) or propidium iodide (Sigma Aldrich). To detect neutrophils, macrophages, DCs, CD4 and CD8 T cells, NK cells and ILCs, cLP cells were stained with antibodies to CD45.2 (104; 0.5 μg/ml), Ly6G (1A8; 2 μg/ml), CD11b (M1/70; 1 μg/ml), CD11c (N418; 1 μg/ml), MHCII (M5/114.15.2; 1 μg/ml), CD64 (X54–5/7.1; 1 μg/ml), F4/80 (BM8; 2 μg/ml), TCRβ (H57–597; 1 μg/ml), NK1.1 (PK136; 2 μg/ml), CD4 (GSK1.5; 1 μg/ml), CD8 (53–5.8; 1 μg/ml), Lineage cocktail (comprising antibodies against TER-119, CD11b, Gr-1, CD3ε and B220; BioLegend) or Thy1.2 (30-H12; 2 μl for 1*10^6^ cells), respectively. To detect ILC subsets among cLP cells, isolated cells were pre-stimulated for 4 h with 50 ng/ml phorbol 12-myristate 13-acetate (PMA; Sigma-Aldrich) plus 2.5 μg/ml ionomycin (Sigma-Aldrich) in the presence of Monensin solution (eBioscience). Prestimulated cells were then stained to detect extracellular markers, including Lineage cocktail (BioLegend). Stained cells were fixed and permeabilized with the Foxp3/Transcription Factor Staining Buffer Set (eBioscience) and stained with antibodies to RORγt (12–6981; 4 μg/ml), T-bet (644,813; 4 μg/ml), GATA3 (25–9966; 4 μg/ml), IL-17a (TC11-18H10.1; 4 μg/ml) and IL-22 (poly5164; 4 μg/ml). To detect M1 macrophages among cLP cells, isolated cells were stained with antibodies to CD45.2, CD11b, CD11c, MHCII, F4/80, CD206 (C068C2; 2 μg/ml) and CD103 (2E7; 1 μg/ml), and stained cells were fixed and permeabilized with Foxp3/Transcription Factor Staining Buffer Set and stained with antibodies to iNOS (ab15323 from Abcam; 4 μg/ml) and Alexa Fluor® 488-conjugated anti-rabbit IgG secondary antibodies (Jackson ImmunoResearch; 7.5 μg/ml). All antibodies were purchased from BioLegend or eBioscience. Cells were acquired on a FACSFortessa instrument (BD Biosciences) and analyzed using the FlowJo cytometric analysis program (Tree Star).

### Purification of innate lymphoid cells

Splenic cells were isolated by mashing spleen through a 40 μm Nylon strainer (BD Biosciences). Red blood cells were removed using ACK lysis buffer (150 mM NH_4_Cl, 1 mM KHCO_3_, 0.1 mM Na_2_EDTA). Single cell suspensions of splenic cells were resuspended in AutoMACS running buffer containing biotin-conjugated anti-mouse Lineage cocktail plus biotin-conjugated anti-mouse NK1.1 and F4/80, and incubated on ice for 20 min. After centrifuge the supernatant was discarded and the splenic cells resuspended in AutoMACS running buffer plus Streptavidin Microbeads (Miltenyi Biotec). Lineage-negative cells were purified using an AutoMACS Separator Pro system (Miltenyi Biotec) and stained with anti-mouse CD45 Microbeads (Milteny Biotec). Lineage-negative, CD45-positive cells were deemed to constitute the ILC subset. Purified innate lymphoid cells (ILCs) were stained with antibodies to CD45.2 (104; 0.5 μg/ml), Thy1.2 (30-H12; 2 μl for 1*10^6^ cells), CD127 (A019D5; 5 μg/ml) antibody and FITC-conjugated streptavidin, and were acquired on a FACSFortessa instrument (BD Biosciences) and analyzed using the FlowJo cytometric analysis program (Tree Star).

### Isolation of peritoneal macrophages and neutrophils

To maximize the yield of peritoneal macrophages, each mouse was injected intraperitoneally with 1 ml of 2.4% thioglycollate 3 days before macrophage harvest. For harvesting, 5 ml PBS was injected into the peritoneum of each euthanized mouse and peritoneal elicited cells (PECs) were collected. After removing red blood cells by ACK buffer treatment, PECs were stained with anti-mouse F4/80 Microbeads (Miltenyl Biotec). F4/80^+^ macrophages were isolated from PECs using an AutoMACS Pro Separator system (Miltenyl Biotec). Isolated F4/80^+^ macrophages were suspended in PBS and keep on ice for further injection. To obtained peritoneal neutrophils for co-incubation experiments, each mouse was injected intraperitoneally with 1 ml of 2.4% thioglycollate 24 h before neutrophils harvest. For harvesting, 5 ml PBS was injected into the peritoneum of each euthanized mouse and PECs were collected. After removing red blood cells by ACK buffer treatment, PECs were stained with anti-mouse Ly6G Microbeads (Miltenyl Biotec). Ly6G^+^ neutrophils were isolated from PECs using an AutoMACS Pro Separator system (Miltenyl Biotec).

### ILC stimulation and cytokines detection by enzyme-linked immunosorbent assay (ELISA)

Isolated splenic ILCs were co-incubated with WT GM-BMDMs at a ratio of 1:1 in 24-well plates (4*10^5^ cells per 400 μl per well) in complete RPMI medium containing 10% FBS and 10 ng/ml each of IL-7 and IL-2 (Preprotech), and supplying with indicated stimuli including 45 ng/ml IFNγ plus 20 ng/ml LPS, or 20 ng/ml IL-4 plus 20 ng/ml IL-13, or 20 ng/ml IL-1β plus IL-23. For accessing IL-22 production by ILC3 is TREM-1(+) macrophages dependent, ILCs were co-incubated with WT or TREM-1-deficient GM-BMDMs in completed medium with or without 5 ng/ml LPS plus plate-bound 8 μg/ml anti-TREM-1 agonist antibody (Thermo) or isotype antibody (Rat IgG2aκ; eBioscience). After 48 h incubation, IL-22 level in supernatant was assessed by specific ELISA (BioLegend) according to the manufacturer’s instructions. For accessing IL-β levels, differentiated WT or TREM-1-deficient GM-BMDMs were incubated in 96-well plates (2*10^3^ cells per 200 μl per well) in complete RMPI medium containing 10% FBS and 45 ng/ml IFNγ plus 20 ng/ml LPS, or 20 ng/ml IL-4 plus 20 ng/ml IL-13, or 5 ng/ml LPS plus plate-bound 8 μg/ml anti-TREM-1 agonist antibody (Thermo) or isotype antibody (Rat IgG2aκ; eBioscience). After 24 h incubation, IL-1β level in supernatant was assessed by specific DuoSet ELISA (R&D) according to the manufacturer’s instructions.

### Statistical analysis

Data from at least two independent experiments were analyzed using GraphPad Prism software, version 6.0 (GraphPad Software). All results were calculated and expressed as the mean ± SEM, and group mean values were evaluated using the Mann-Whitney nonparametric test or the unpaired t test with Welch’s correction. For all statistical analyses, statistical significance is indicated by *, *p* < 0.05; **, *p* < 0.01 or ***, *p* < 0.001 for comparison between DSS-treated WT and DSS-treated TREM-1 KO group, and #, *p* < 0.05; ##, *p* < 0.01 or ###, *p* < 0.001 for comparison to the mock group.

## Results

### TREM-1 deficiency exacerbates DSS-induced colitis

**Fig. 1:**
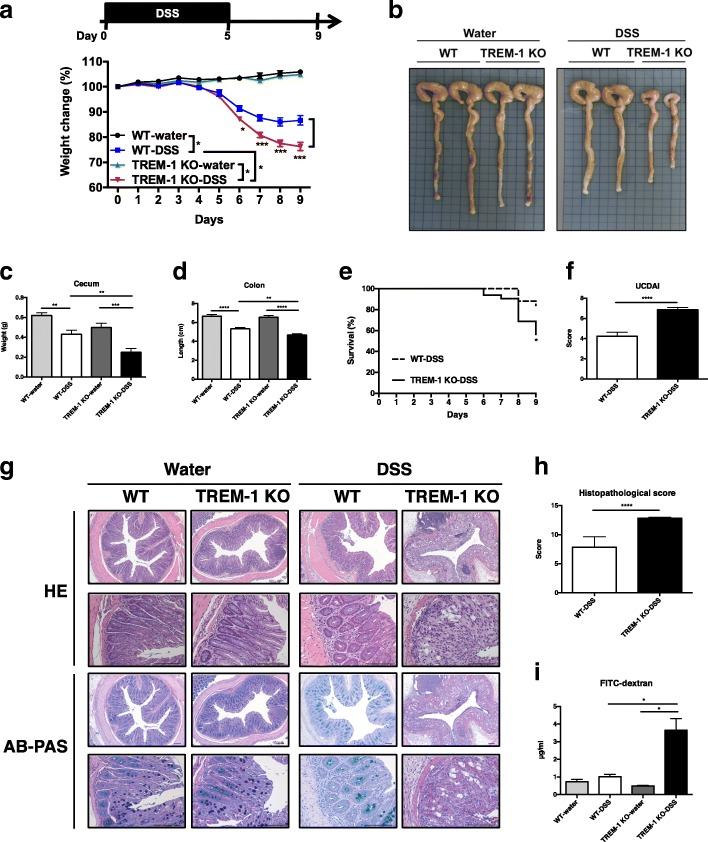
TREM-1 deficiency exacerbates DSS-induced colitis in mice. WT and TREM-1 KO mice (*n* = 27/group) were supplied with normal drinking water or drinking water containing 3% DSS for 5 days, followed by normal drinking water for 4 days. **a** Weight change, **b** images of colon length and cecum size (one square = 1 cm), **c** cecum weight, **d** quantitation of colon length, **e** survival rate, and **f** UCDAI score were all determined on day 9. Data in a, c, d, and f are the mean ± SEM. *P* values of differences were determined by Mann-Whitney nonparametric test. *, *p* < 0.05; **, *p* < 0.01; ***, *p* < 0.001 or ****, *p* < 0.0001. Survival rate was compared using the log-rank test, *, *p* < 0.05. **g** Representative images of histopathology of sections of colonic tissues that were prepared from water-treated WT and TREM-1 KO mice (*n* = 17/group) and DSS-treated WT and TREM-1 KO mice (*n* = 27/group) and stained with HE or AB-PAS. Data are representative of 7 independent experiments. **h** Histopathological scores of the DSS-treated mice in (**g**). Data are the mean ± SEM. **i** Epithelial barrier permeability as determined by measuring FITC-dextran levels in the serum of the WT and TREM-1 KO mice in (**g**) on day 9 after DSS initiation. Data are pooled from two independent experiments involving 6 mice per group and are the mean ± SEM

Previous studies have demonstrated that increased expression of TREM-1 on colonic macrophages is an important indicator of colonic inflammation in both mice and humans [12]. Inactivation of TREM-1, either by administering antagonist peptides or by engineering depletion, attenuates the severity of chemically induced experimental colitis [12, 31]. These observations prompted us to first assess whether our in-house generated TREM-1 deficient (TREM-1 KO) mice were also protected from DSS-induced colitis. Acute colitis was initiated by daily administration of 3% DSS in drinking water for 5 days (induction phase) followed by 4 days of regular tap water (recovery phase). Age-matched male C57BL/6 (WT) and TREM-1 KO mice subjected to this protocol all exhibited weight loss and signs of loose stool or diarrhea with rectal bleeding as early as 3 days post-DSS initiation (Fig. 1a). By day 5, all WT and TREM-1 KO mice had lost 2–5% of their initial body weights. However, by day 9 of the DSS protocol (the humane endpoint), TREM-1 KO mice had shown a significantly greater degree of weight loss (20–30%) compared to WT controls as well as a greater degree of dehydration and diarrhea (Fig. 1a). Once tap water was administered on day 5 of the DSS protocol, the intestinal epithelium of WT mice started to recover its intestinal barrier function, and most WT mice began to ameliorate body weight loss on day 7 (Fig. 1a). However, to our surprise, TREM-1 KO mice continued to exhibit dramatic weight loss after day 7. The colons of the TREM-1 KO mice were significantly shortened, and the ceca of the knockout mice were also decreased in size and barely filled with fecal content on day 9 (Fig. 1b-d). Mortality was consequently increased, and no TREM-1 KO mouse was able to recover from DSS-induced acute colitis over days 5–9 (Fig. 1e). The above pathologies contributed to the significantly increased scores for the ulcerative colitis disease activity index (UCDAI) in TREM-1 KO mice (Fig. 1f). In addition, representative histological sections of colons from DSS-treated TREM-1 KO mice exhibited prominent crypt and goblet cell loss that exceeded those in DSS-treated WT mice. A histopathology score composed of evaluations of immune cell infiltration, epithelial injury, crypt damage and percentage of area damaged was also increased in the knockout mice (Fig. 1g-h). These data suggested that TREM-1 deficiency reduced the integrity of the intestinal epithelial barrier, enhancing the tissue damage caused by DSS treatment. We next assessed intestinal barrier permeability by applying FITC-conjugated dextran to the colon intrarectally and measuring the amounts of this agent leaking from the intestinal lumen into the blood within 30 min. Intestinal barrier permeability in TREM-1 KO mice appeared normal prior to DSS treatment, but FITC-dextran levels rose significantly in the serum of TREM-1 KO mice by day 9 post-DSS initiation (Fig. 1i). Taken together, these data demonstrate that TREM-1 KO mice have lost the ability to rapidly reconstitute the integrity of the intestinal barrier after the damage-inducing chemical DSS is removed.

### TREM-1 increases production of IL-22 by ILC3 cells in the colons of DSS-treated mice

**Fig. 2:**
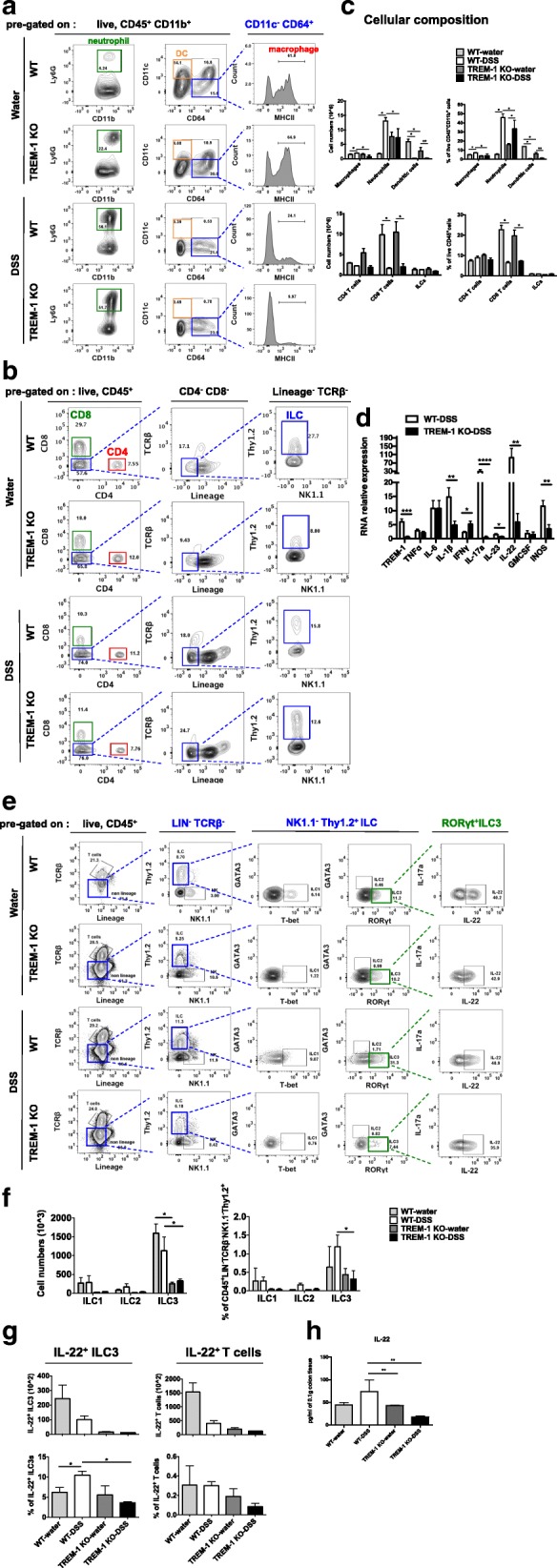
Depletion of TREM-1 affects neutrophil and macrophage infiltration, inflammatory cytokines induction and decreases IL-22 production by ILC3 cells in colons upon DSS treatment. **a-b** Contour plot of FACS analysis data showed the gating strategy employed to identify neutrophils, DCs, CD4 T cells, CD8 T cells and ILC cells in colons from water- or DSS-treated WT and TREM-1 KO mice (*n* = 10/group) on day 7 post-DSS initiation. Lamina propria macrophages were subcategorized into CD45^+^CD11b^+^CD11c^−^CD64^+^ and further presented by histogram of MHCII staining. **c** FACS analysis of the percentage and absolute number of the indicated immune cells among colonic cells from the indicated mice in (**a**-**b**). Data are the mean ± SEM and representative of at least three independent experiments. **d** Real-time PCR analysis of mRNA levels of the indicated genes in colonic tissue of DSS-treated WT or TREM-1 KO mice on day 9. Data are presented as fold change relative to the GAPDH mRNA level and are representative of at least 3 independent experiments involving 20 mice per group. **e** Contour plot of FACS analysis data showed the gating strategy employed to identify distinct ILC subsets by intracellular staining with specific transcription factors. **f** FACS analysis of the frequency and absolute number of the indicated ILC subsets among live CD45^+^LIN^−^TCRβ^−^NK1.1^−^Thy1.2^+^ cells isolated from colons of water-treated WT and TREM-1 KO mice and DSS-treated WT and TREM-1 KO mice (*n* = 8/group) on day 7 post-DSS initiation. **g** The percentage and absolute number of CD45^+^LIN^−^TCRβ^−^NK1.1^−^Thy1.2^+^RORγt^+^IL-22^+^ ILC3 cells and CD45^+^TCRβ^+^IL-22^+^ T cells among colonic cells from the indicated mice (*n* = 10/ group) on day 7 post-DSS initiation. Data are the mean ± SEM and are representative of 3 independent experiments. **h** Quantitation of IL-22 as determined by ELISA in 0.1 g of colonic tissue from water-treated WT and TREM-1 KO mice (*n* = 5/group) and DSS-treated WT and TREM-1 KO mice (*n* = 5/group) on day 7 post-DSS initiation. Data are mean ± SEM and representative of two independent experiments. Statistical significance was determined by Mann-Whitney nonparametric test. * *p* < 0.05 or **, *p* < 0.01as indicated

Disruption of the colonic epithelium by DSS allows luminal microbes to penetrate into the colonic lamina propria (cLP), leading to acute inflammation. A common feature of this acute colitis is the massive infiltration of innate immune cells that produce large amounts of pro-inflammatory mediators such as TNFα, IL-6, IL-1β, IL-23 and GMCSF. In this light, we analyzed changes in immune cell compartments at day 7 and RNA levels of pro-inflammatory cytokines at day 9 in the colons of our WT and TREM-1 KO mice after DSS initiation. Viable CD45^+^ immune cells were analyzed by flow cytometry and subcategorized into individual subsets according to their expression of common cell surface markers, as follows: CD11b^+^CD11c^−^CD64^+^MHCII^+^, macrophages; CD11b^+^Ly6G^+^, neutrophils; CD11b^+^CD64^−^CD11c^+^, dendritic cells (DCs); CD4^+^ T cells; CD8^+^ T cells and Lin^−^TCRβ^−^NK1.1^−^Thy1.2^+^, ILCs (Fig. 2a-b). Consistent with previous reports, the percentage and absolute number of macrophage and neutrophil were significantly increased in our WT mice compared to TREM-1 KO mice due to DSS treatment (Fig. 2c). Intriguingly, CD8 T cell and DC were both decreased in the frequency and cell numbers in the colons of DSS-treated mice (Fig. 2c). However, CD4 T cell and ILC showed no significant changes in the colons of DSS-treated mice (Fig. 2c). The reducing infiltrating macrophages and neutrophils, which was unexpected due to our initial observation of exacerbated colitis in TREM-1 KO colons. This result led us to investigate the inflammatory cytokine profile of our TREM-1 KO mice.

To determine the levels of various cytokines known to aggravate DSS-induced colitis, we used real-time PCR to screen colons from DSS-treated WT and TREM-1 KO mice for mRNA levels of inflammatory cytokines. Previous reports have demonstrated a role for TREM-1 in amplifying pro-inflammatory cytokine production and oxidative burst [32]. When we analyzed colon tissues from our WT and TREM-1 KO mice at day 9 post-DSS initiation, we found that, as expected, WT colons showed significant increases in TREM-1, IL-1β, IL-17a, IL-23, IL-22 and inducible nitric oxide (iNOS) compared to TREM-1 KO colons (Fig. 2d). However, levels of TNFα, IL-6 and GMCSF mRNAs were comparable in treated WT and TREM-1 KO colons (Fig. 2d). These data indicate that the DSS-mediated tissue damage observed in mice lacking TREM-1 cannot simply be explained by the enhanced immune cell infiltration and inflammatory mediator production observed in affected colons.

Recent studies have implicated IL-22-producing ILC3 cells present in the intestinal barrier as playing an important role in regulating intestinal homeostasis, inflammation and host protection [15, 20]. Indeed, UC patients show significantly reduced numbers of IL-22^+^ cells in actively inflamed tissues [24]. In addition, clinical evidence has suggested that IL-22 derived from RORγt^+^ ILCs can help to prevent UC onset [15]. We next examined whether the tissue damage in treated TREM-1 KO colons could be due to an impairment of epithelial layer repair, which may in line with reduce IL-22-producing ILC3 cells. IL-1β and IL-23 are potent drivers of ILC3 activation and IL-22 production, which enhance innate immune defenses in mucosal tissues [24]. Importantly, we found that mRNA levels of IL-1β, IL-23, IL-17a and IL-22 were all significantly decreased in TREM-1 KO mice colons (Fig. 2d). To further confirm that DSS-treated TREM-1 KO mice suffer intestinal barrier impairment due to loss of ILC3 effector function, we analyzed the percentage of IL-22-producing RORγt^+^ ILC3 in colons of WT and TREM-1 KO mice on day 7 post-DSS initiation. ILC subsets were identified by CD45^+^LIN^−^TCRβ^−^NK1.1^−^Thy1.2^+^ cells with specific transcription factors (Fig. 2e). The percentage and absolute number of T-bet^+^ ILC1 and GATA3^+^ ILC2 were comparable in the colons of WT and TREM-1 KO mice (Fig. 2f). RORγt^+^ ILC3 showed no differences in the frequency and absolute number of WT DSS-treated colons compared to WT water-treated colons; by contrast, the frequency and absolute numbers of ILC3 was dramatically reduced in TREM-1 KO DSS-treated colons compared to WT (Fig. 2f). In line with our hypothesis, loss of TREM-1 significantly reduced the percentage of IL-22 in ILC3 cells but not in T cells in DSS-treated colons, as determined by intracellular FACS staining (Fig. 2g), and levels of IL-22 protein in these colonic tissues were also markedly decreased (Fig. 2h). These data reveal a novel role for TREM-1 in regulating intestinal barrier integrity in response to DSS by promoting ILC3 cell activation.

### TREM-1-mediated M1 macrophage polarization is crucial for IL-22 production by ILC3 cells

**Fig. 3:**
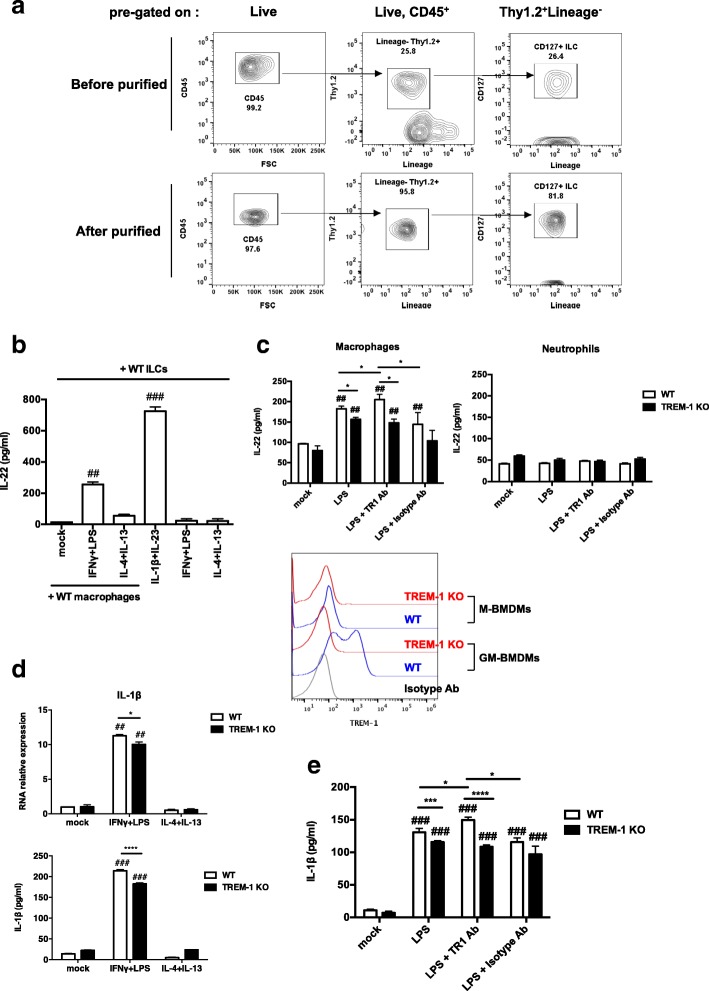
TREM-1-mediated IL-1β induction by M1 macrophages elicits IL-22 production by ILC3. **a** Contour plot of FACS analysis data showed the purity of AutoMACS-purified ILCs **b** ELISA determination of IL-22 production by AutoMACS-purified WT Lin^−^CD45^+^ ILCs that were co-cultured with WT GM-BMDMs in the presence or absence of the indicated murine recombinant cytokines. Culture supernatants were harvested after 48 h. Mock, no treatment. ILCs treated with IL-1β + IL-23 in the absence of macrophages served as positive control and IFNγ + LPS or IL-4 + IL-13 in the absence of macrophages served as negative control. Data are the mean ± SEM and representative of four independent experiments performed in triplicate. **c** ELISA determination of IL-22 production by AutoMACS-purified WT Lin^−^CD45^+^ ILCs that were co-cultured with WT or TREM-1 KO cells (GM-BMDMs or neutrophils, respectively) in the presence of LPS or LPS plus anti-TREM-1 agonistic antibodies (TR1 Ab) or isotype antibodies, respectively. Data are mean ± SEM and representative of 3 independent experiments performed in triplicate. Histogram of FACS analysis data showed TREM-1 levels on M- or GM- BMDMs. **d-e** Real-time PCR analysis of mRNA level of IL-1β and ELISA determination of IL-1β production by WT- or TREM-1 KO- GM-BMDMs that were cultured in the presence of the polarizing cytokines (IFNγ + LPS for M1 or IL-4 + IL-13 for M2). Culture supernatants were harvested after 24 h. Mock, no treatment. For (**b-e**), for comparisons between WT and TREM-1 KO, differences were evaluated using an unpaired t test with Welch’s correction: *, *p* < 0.05; **, *p* < 0.01; ***, *p* < 0.001 or ****, *p* < 0.0001. For comparison to the mock group, differences were evaluated using an unpaired t test with Welch’s correction: #, *p* < 0.05, ##, *p* < 0.01 or ###, *p* < 0.001

Intestinal macrophage drives ILC3 activation and its IL-22 production by secreting IL-1β. It is known that M1 macrophage produces high amounts of pro-inflammatory cytokines such as TNFα, IL-6 and IL-1β. Whether macrophage plasticity and their cytokine profile affects ILC activation remains to be determined. We therefore evaluated the contributions of M1 and M2 macrophages to the regulation of ILC production of IL-22. We enriched Lin^−^CD45^+^ ILCs from WT spleens using an AutoMACS Pro Separator, examined the purity of AutoMACS-purified ILCs were analyzed by FACS staining (Fig. 3a), and then co-cultured the isolated ILCs with or without WT GM-BMDMs under polarizing conditions: IFNγ + LPS to induce M1 differentiation, and IL-4 + IL-13 to induce M2. IL-22 levels in the culture supernatants were determined after 48 h of incubation. The positive control for IL-22 production was a culture of WT ILCs incubated with IL-1β + IL-23. The effect of polarizing cytokines on ILC themselves was also determined (Fig. 3b). Intriguingly, WT macrophages that were stimulated under M1 conditions induced IL-22 production by ILC3 much more strongly than did non-stimulated or M2 macrophages (Fig. 3b). Thus, our data suggest that M1 macrophages support ILC3 production of IL-22.

To examine whether macrophage-induced ILC3 production of IL-22 is TREM-1-dependent, Lin^−^CD45^+^ ILCs enriched from WT spleens were co-cultured with GM-BMDMs from WT or TREM-1 KO mice in the presence or absence of low dose LPS (5 ng/ml) or cross-linking anti-TREM-1 agonist antibodies (8 μg/ml). Due to TREM-1 is preferentially expressing on GM-BMDMs compared to MCSF-derived BMDMs (M-BMDMs), we used GM-BMDMs to co-incubate with splenic ILCs for further study (Fig. 3c). Consistent with the importance of M1 macrophages in supporting IL-22 production, WT ILCs cultured with LPS-stimulated WT GM-BMDMs significantly increased their IL-22 production compared to ILCs cultured with LPS-stimulated TREM-1 KO GM-BMDMs (Fig. 3c). Moreover, these enhanced IL-22 levels were boosted by anti-TREM-1 antibody engagement compared to the isotype control antibody (Fig. 3c). TREM-1 is also highly expressed on neutrophils; however, IL-22 production by ILC3 was not induced by culturing with neutrophils (Fig. 3c). TREM-1 promoted M1 macrophage to produce more IL-1β in WT GM-BMDMs compared to TREM-1 KO, moreover, TREM-1 engagement also boosted IL-1β secretion by LPS-stimulated WT GM-BMDMs compared to LPS-stimulated TREM-1 KO GM-BMDMs (Fig. 3d-e). Collectively, these results indicate that inflammatory macrophages are crucial for regulating ILC3 activity and suggest that TREM-1-mediated M1 macrophage polarization and IL-1β secretion enhance IL-22 production by ILC3.

### TREM-1 mediates M1 macrophage polarization in line with inflammatory cytokine induction in DSS-treated colons

**Fig. 4:**
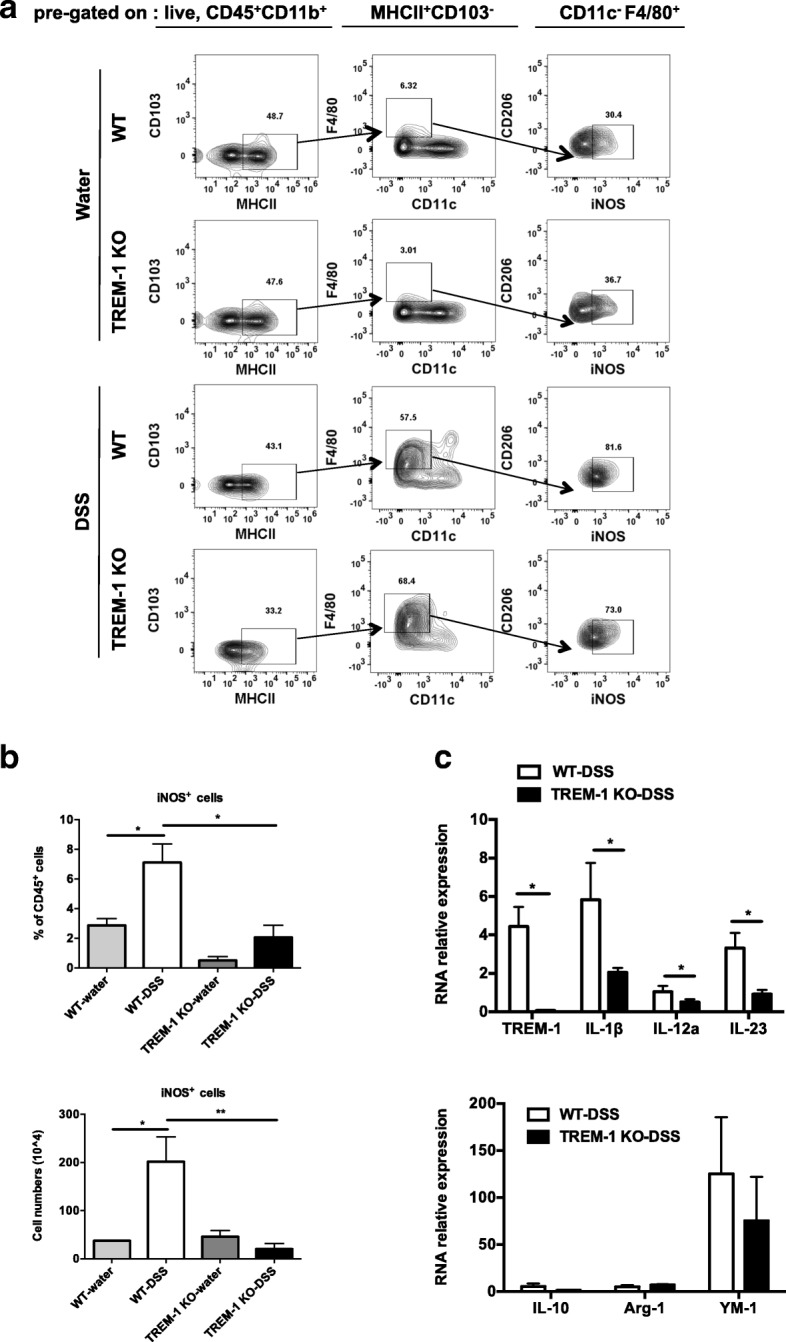
TREM-1 promotes M1 macrophage polarization in line with increased M1 cytokine profile but not M2 in DSS-treated colonic macrophages. Lamina propria macrophages were isolated from colons of water- or DSS-treated WT and TREM-1 KO mice (*n* = 6/group) on day 7 post-DSS initiation. **a** Contour plot of FACS analysis data showed the gating strategy employed to identify distinct iNOS^+^ M1 macrophages among CD45^+^CD11b^+^MHCII^+^CD103^−^CD11c^−^F4/80^+^ cells. **b** Percentage and absolute number of iNOS^+^ macrophages as determined by intracellular staining in (**a**). Data are the mean ± SEM of two experiments. *, *p* < 0.05 or **, *p* < 0.01 by Mann-Whitney nonparametric test. **c** Real-time PCR analysis of mRNA levels of the indicated genes in the AutoMACS-purified F4/80^+^ colonic macrophages from the DSS-treated mice. Data are the mean ± SEM of two experiments and are expressed as fold change relative to GAPDH mRNA. *, *p* < 0.05 by Mann-Whitney nonparametric test

iNOS production is a signature feature of M1 macrophages [33]. We previously showed that agonistic anti-TREM-1 antibody treatment strengthens the induction of iNOS in M1 macrophages and enhances their ability to produce pro-inflammatory cytokines [7]. We therefore speculated that TREM-1 might also regulate macrophage plasticity in the cLP during DSS-induced colitis. To test this hypothesis, we isolated cLP cells from the colons of WT and TREM-1 KO mice at day 7 post-DSS initiation and analyzed M1 polarized macrophages among viable CD45^+^CD11b^+^CD103^−^MHCII^+^CD11c^−^F4/80^+^ cells by FACS staining (Fig. 4a). As expected, the percentage and absolute number of iNOS^+^M1 macrophages were significantly increased in inflamed WT colons compared with DSS-treated TREM-1 KO colons (Fig. 4b). In addition, these WT macrophages expressed significantly increased levels of mRNAs for not only TREM-1 but also inflammatory cytokines such as IL-12a, IL-1β and IL-23 (Fig. 4c). In addition, our data showed that the levels of M2 relative cytokines were comparable in DSS-treated colons of WT and TREM-1 KO mice (Fig. 4c). These data support our contention that TREM-1 mediates M1 polarization of intestinal macrophages in vivo and upregulates IL-1β. Because IL-1β is a potent inducer of IL-22 production by ILC3 cells, these results suggest a novel mechanism by which TREM-1 can protect the intestine from DSS-induced epithelial barrier impairment.

### Exogenous delivery of IL-22 or TREM-1-expressing WT macrophages can protect TREM-1-deficient mice against DSS-induced colitis

**Fig. 5:**
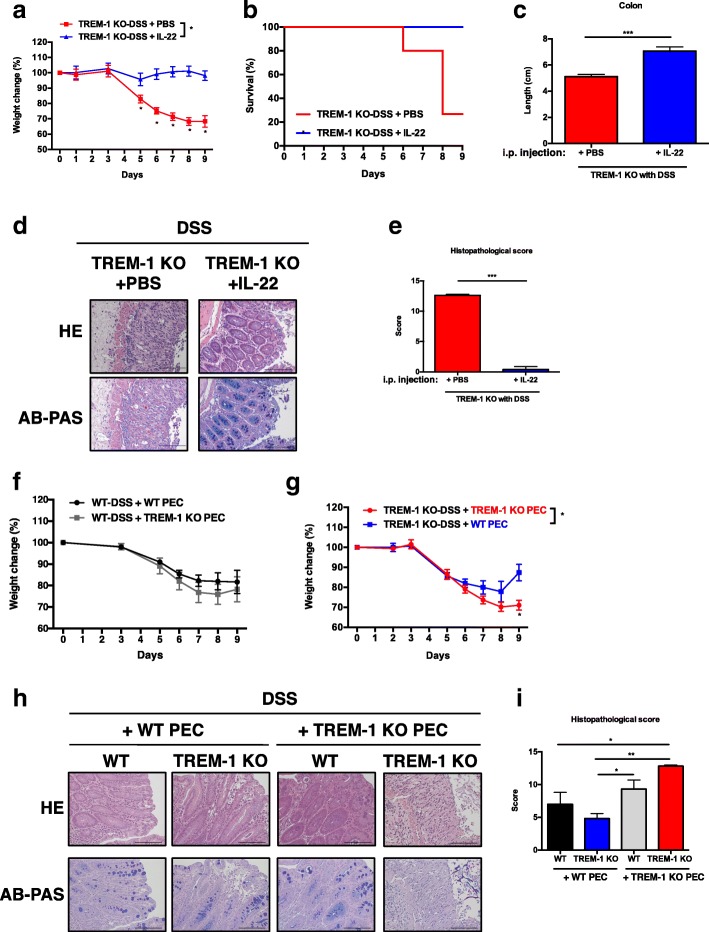
Treatment with exogenous IL-22 or TREM-1-expressing macrophages rescues TREM-1-deficient mice from DSS-induced colitis. WT and TREM-1 KO mice were intraperitoneally injected with 500 ng IL-22 in PBS, or PBS alone, on every other day starting 1 day before (day − 1) initiating the standard DSS protocol. **a** Weight change, **b** survival rate, **c** colon length, **d** representative HE and AB-PAS staining, and **e** histopathological score were determined in DSS-treated WT mice, PBS + DSS-treated TREM-1 KO mice, and IL-22 + DSS-treated TREM-1 KO mice (*n* = 8/group). Data in **a**, **c** and **e** are the mean ± SEM and representative of two independent experiments. Statistical significance was determined by Mann-Whitney nonparametric test. *, *p* < 0.05; **, *p* < 0.01 or ***, *p* < 0.001 as indicated. Survival rate was compared using the log-rank test, *, *p* < 0.05. **f-g** Weight changes in (**f**) WT mice (*n* = 6/group) and (**g**) TREM-1 KO mice (*n* = 6/group) that were treated with DSS and intraperitoneally injected with by AutoMACS-purified F4/80^+^ peritoneal elicited macrophages (PECs) isolated from either WT or TREM-1 KO mice, as indicated. Data are mean ± SEM and representative of two independent experiments. **h** Representative images of histopathology of sections of colonic tissues that were prepared from DSS-treated WT and TREM-1 KO mice with intraperitoneally injected either WT or TREM-1 F4/80^+^PECs and stained with HE or AB-PAS. Scale bar: 100 μm. **i** Histopathological score were determined in DSS-treated WT mice with WT or TREM-1 KO F4/80^+^PECs adoptive transferred, and DSS-treated TREM-1 KO mice with WT or TREM-1 KO PECs adoptive transferred, respectively (*n* = 6/group)

Our data above showed that mice lacking TREM-1 suffer from impaired intestinal integrity due to a defect in ILC3 cell activation which compromises IL-22 production. To verify the importance of IL-22 in preventing the pathogenesis of DSS-induced colitis, we injected recombinant IL-22 into the peritoneal cavities of TREM-1-deficient mice every other day, starting 1 day prior to DSS administration. IL-22 treatment resulted in a striking attenuation of weight loss in DSS-treated TREM-1 KO mice compared to PBS-treated controls (Fig. 5a). The increased mortality and shortened colons observed in DSS-treated TREM-1 KO mice were also reversed by IL-22 (Fig. 5b-c). Histopathological analysis revealed that IL-22 administration prevented morphological changes in the intestinal barrier such as crypt damage, goblet cell loss, and deep tissue injury (Fig. 5d-e). Thus, exogenous IL-22 protected TREM-1 KO mice from developing intestinal damage during DSS-induced acute colitis. Importantly, adoptive transfer of WT macrophages into DSS-treated TREM-1 KO mice significantly reduced colitis and tissue damage in comparison to TREM-1 KO mice that received TREM-1 KO macrophages (Fig. 5f-i). Taken together, these results demonstrate that TREM-1 expression by macrophages is crucial for maintaining the integrity of the intestinal epithelium under chemical threat and that this protection is mediated by an IL-22-dependent pathway.

## Discussion

Previous studies using experimental mouse colitis models have shown that TREM-1 deficiency alleviates IBD by reducing the production of inflammatory mediators [12, 31]. However, precisely how TREM-1 deficiency mediates its protective role against colitis was not delineated. Here, we have generated an independent TREM-1 KO mouse line to unambiguously investigate TREM-1’s function during DSS-induced colitis. As expected, our DSS-treated TREM-1-deficient mice exhibited reductions in innate cells infiltration and inflammatory mediator production compared to DSS-treated WT mice. Surprisingly, however, despite this decrease in inflammatory mediators, intestinal tissue damage still occurred in the absence of TREM-1. It was previously reported that TREM-1 is essential for restricting *Klebsiella pneumoniae* translocation across the intestinal epithelium, implying that TREM-1 may participate in regulating intestinal barrier function [34]. In line with this finding, our results demonstrate that TREM-1 indeed plays a dual role in protecting the intestinal epithelium. Not only does TREM-1 significantly boost the production of IL-1β, IL-23, IL-17 and iNOS following DSS treatment, thereby helping to defend the intestine from any incipient invasion of luminal microbes, but TREM-1 also promotes the repair of the intestinal epithelium by activating IL-22 production by ILC3 cells.

In a previous study by Weber et al., although mouse weight loss was attenuated by TREM-1 deficiency, there were no obvious changes in pro-inflammatory mediators. It should be noted that the mice in Weber’s study have differed in their intestinal microflora from the mice in our study, since the former were maintained in a *Helicobacter*-negative environment prior to the initiation of DSS-induced colitis. By contrast, our mice were maintained in a *Helicobacter*-positive environment, a relevant factor because an association between *Helicobacter spp.* infection and colitis pathogenesis has been reported [35, 36]. Whether the differences between the Weber study and our work are due to differences in *Helicobacter spp*. intestinal colonization remains to be further investigated.

Previous reports have indicated that colonic macrophages serve a protective role in DSS-induced colitis [5]. Intestinal macrophages, such as CX_3_CR1^+^ macrophages in mice and CD14^+^ monocytes in human, have been shown to regulate the production of IL-22 by ILC3 cells [24]. DSS disrupts the intestinal barrier, and the mucosal microenvironment in the inflamed colon may drive the inflammatory differentiation of macrophages. However, the effects of macrophage plasticity on ILC activation have yet to be completely defined. We found that anti-TREM-1 antibody engagement synergistically amplified LPS-induced IL-1β production, which is consistent with our observations in DSS-treated colons and with our hypothesis that inflammatory macrophages drive ILC activation in a TREM-1-dependent manner. A recent study has demonstrated that colon-infiltrating neutrophils produce IL-22 in response to coordinated signaling by IL-23 and TNFα, suggesting a role for granulocytes in supporting enhanced epithelial barrier function [16]. However, although we did observe significantly increased neutrophil numbers in inflamed colons, these cells did not produce IL-22. In addition, unlike macrophages, neutrophils co-cultured with ILCs do not induce these cells to produce IL-22 in response to LPS or anti-TREM-1 agonistic antibody. Thus, we believe that the IL-22 responsible for intestinal barrier integrity is secreted solely by ILC3 and not by granulocytes.

Although TREM-1 deficiency has been shown to exhibit reduced renal pathology, conversely, TREM-1 deficiency exacerbates the disease activity of microbial-induced sepsis, liver abscesses, and lupus [7, 8, 34, 37]. The impact of TREM-1 deletion on different inflammatory diseases is still controversial. In order to further discuss the differences between WT and TREM-1 KO macrophages, we screened gene profile by RNA sequencing (RNAseq). RNAseq analysis of GM-BMDMs revealed an unexpected TREML4 induction in TREM-1 KO relative to WT controls during basal conditions (data not shown). However, this elevation in TREML4 expression was not observed in GM-BMDMs from other TREM-1 transmembrane deletion (exon 3 deletion in *Trem1* gene by using CRISPR/Cas9) mice using an independent targeting strategy (data not shown). A previous report shows that TREM-2 has no impact on early time points after systemic central nerve inflammation due to an overwhelming increase in TREML1 (which is an adjacent gene directly located the downstream of TREM-2) in TREM-2 deficient mice [38]. However, removal of the floxed neomycin cassette in a specific TREM-2 KO mouse line completely prevented the TREML1 overexpression artifact [38]. Of note, Weber et al. generated a constitutive TREM-1 deficient mouse with PuroR and Neomycin cassette deletion [31]. These finding might delineate the controversial pathologies of DSS-induced colitis, which are observed in the specific TREM-1 KO mouse lines from two different groups. Therefore, it needs to be elucidated whether unexpected TREML4 upregulation in TREM-1 KO GM-BMDMs is due to the remaining neomycin cassette, thereby masking the impact of TREM-1 deficiencies in various animal models. Additionally, TREM-1 deletion not only alters TREML4 expression depending on the targeting construct, but an unexpected truncated TREM-1 transcript and protein (with exon 2 deletion) are present in TREM-1 KO GM-BMDMs as well (data not shown). It is therefore uncertain as to whether aberrant TREM-1/TREML4 gene expression arise disease activity in TREM-1 KO mice upon DSS treatment.

In summary, our findings indicate that TREM-1 protects mice against acute DSS-induced colitis by promoting M1 macrophage polarization and IL-1β production, which contributes to IL-22 production by ILC3 that restores epithelial barrier integrity.

## Conclusion

Our findings indicate that TREM-1 protects mice against acute DSS-induced colitis by promoting ILC3 activation and the production of IL-22 that restores epithelial barrier integrity. This support of ILC3-mediated IL-22 synthesis is due to a novel function of TREM-1: the promotion of M1 macrophage polarization and IL-1β production.

## Data Availability

Data and materials related to this study are available upon request.
